# Workplace Health Promotion: Assessing the Cardiopulmonary Risks of the Construction Workforce in Hong Kong

**DOI:** 10.1371/journal.pone.0146286

**Published:** 2016-01-22

**Authors:** Sze Pui Pamela Tin, Wendy W. T. Lam, Sungwon Yoon, Na Zhang, Nan Xia, Weiwei Zhang, Ke Ma, Richard Fielding

**Affiliations:** Division of Behavioural Sciences, School of Public Health, 5/F William MW Mong Block, 21 Sassoon Road, Pokfulam, The University of Hong Kong, Hong Kong SAR, China; Columbia University, UNITED STATES

## Abstract

**Objective:**

Health needs of different employee subgroups within an industry can differ. We report the results of a workplace cardiopulmonary risk assessment targeting workers and support staff in the construction industry.

**Methods:**

A free worksite-based cardiopulmonary risk assessment for 1,903 workers on infrastructural contracts across Hong Kong was initiated in May 2014. Cardiopulmonary risk screening was performed in 60-minute blocks for approximately 30 workers/block with individualized feedback and lifestyle counseling. Risk profiles stratified by occupational roles are differentiated using the χ^2^-test for categorical and Student’s t-test for continuous variables.

**Results:**

Most construction workers and clerks/professionals were male (83.2% and 71.2%, respectively) and Chinese (78.7% and 90.9%, respectively). Construction workers were older (mean: 44.9 years, SD 11.5) and less well-educated (6.1% received tertiary education) than clerks/professionals (35.0 years, 10.7; 72.6% received tertiary education), but more likely to be hypertensive (22.6% vs. 15.4%, *p*<0.001), overweight/obese (71.7% vs. 56.6%, *p*<0.001), centrally obese (53.1% vs. 35.5%, *p*<0.001), and have undesirable levels of high-density lipoprotein (41.6% vs. 35.8%, *p*<0.05) and diabetic levels of non-fasting blood glucose (4.3% vs. 1.6%, *p*<0.05). Up to 12.6% of construction workers and 9.7% of office clerks/professions had three or more metabolic syndrome risk factors. While construction workers were more likely than clerks/professionals to be daily smokers, they reported better work-related physical activity and diet.

**Conclusions:**

Simple worksite health risk screening can identify potentially high-cardiopulmonary-risk construction industry employee subgroups for onward confirmatory referral. Separate cardiopulmonary health promotion strategies that account for the varying lifestyle profiles of the two employee subgroups in the industry appear justified.

## Introduction

Workplaces provide opportunities for health risk assessment and promotion initiatives that have the potential for reaching out to large working populations [1, 2]. Such initiatives can help to reduce employees’ chronic diseases risk factors [3, 4], contribute to safeguarding their health and hence, their productivity [2, 5]. Importantly, workplace initiatives can provide valuable encouragement or triggers for employees who otherwise do not undergo health checks elsewhere. This is particularly relevant to workers in the construction industry who may be less educated and knowledgeable about health risks [2].

Rapid infrastructure expansion globally coupled with difficult employment and working conditions places the construction industry worker force at risk. Occupational injuries and musculoskeletal problems among construction workers are common, negatively impact upon work performance and contribute to sickness absence [6, 7], prompting emphasis on the promotion of occupational safety. However, although poor cardiovascular health also significantly contributes to sickness absence [7], the cardiovascular health of construction workers is easily overlooked as a negative culture towards physical health promotion generally exists within the industry [8]. When considering the physically demanding nature of construction work that makes cardiopulmonary fitness a priority among the workers, poor cardiopulmonary health becomes problematic, particularly in an aging workforce that is more prone to health problems. Additionally, pollution and dust coupled with the need to engage in underground work puts employees of construction industry at risk for compromised pulmonary health.

Known to be uniquely demanding and highly stressful [9], the Hong Kong construction industry now faces an outflow of workers to projects in Mainland China, Macau and elsewhere that offer more competitive salaries and job opportunities [10]. This and the aging construction workforce that has failed to attract younger workers [10–13] means that the local construction industry is now facing a serious manpower shortage [10, 11]. Maximizing the productivity among the existing workforce and lessening the number days taken off work through health preservation is thus a priority, making the local construction workforce a unique target population for health assessment and promotion initiatives.

A worksite cardiopulmonary health risk assessment and promotion intervention should contribute to safeguarding the health of the local construction workforce, especially since the cardiopulmonary health profiles of local construction workers are currently unknown. While generalized information is much less likely to prompt behavior change than is more personalized information, a tailored health needs assessment would be too expensive and time consuming. One alternative is a simple “quick and clean” screening to pick up people with potential risk markers for onward referral to their own health providers or worksite occupational health clinics. This study reports the results of a workplace cardiopulmonary risk assessment targeting the construction workforce involved in major local railway infrastructure developments. While workplace interventions generally target all employees within an occupational setting, the differing work nature of employees from different segments of the same industry implies dissimilar health risks and needs that calls for different health interventions [14]. Given the stark contrast in the physically demanding work of construction workers and the relatively sedentary nature of work that the construction industry office clerks are engaged in, the construction workforce provides a good opportunity to explore any differences in needs. Hence, apart from reporting the risk profiles of construction workers, we compared profiles of construction workers with those of office clerks/professional colleagues. The lifestyle characteristics of employees were also profiled and reported.

## Methods

### Procedure

The ‘Worker Health and Wellbeing Month’ (WHM) was initiated by the MTR Corporation Limited (MTR) in May 2014 as part of the Hong Kong government’s 2014 health and safety month designed and implemented by a team commissioned from the Division of Behavioural Sciences, School of Public Health at The University of Hong Kong. The aim was to provide free on-site cardiopulmonary risk profiling for sub-contracted employees working on five major railway infrastructure contracts throughout Hong Kong during working hours. While the theme of the WHM was “cardiovascular health”, we also screened pulmonary function.

Participant availability was determined by the employing contractors at different construction sites according to work schedules. Since it is unlikely that contractors would have purposefully rearranged work schedules to allow the participation of certain workers, worker release did not follow any clear patterns that we were aware of and sampling bias is thus minimized. Despite careful scheduling of workers groups, each of 30 workers, there was disordered arrival and flow of work groups due to various reasons including work schedule delays, poor weather and staggered breaks. The two major participating employee subgroups included construction workers (frontline skilled or unskilled (labourer) tradesmen working at the infrastructure worksite) and office clerks/professionals (clerical staff, architects, engineers, surveyors, management level staff, safety officers and technicians.

### Measures

A total of 71 one-hour health screening and promotion sessions were implemented at 37 different worksites (pre-selected by the MTR). Each one-hour WHM session assessed one worker group. Each worker rotated through each assessment station in a quasi-random sequence determined by station vacancies. Assessment stations measured a) blood pressure (systolic and diastolic, mmHg), blood glucose (mmol/L) and whole blood cholesterol (total and high density lipoprotein (HDL), mmol/L) (three stations, each measuring all three indices); b) body mass index (BMI) and waist-to-hip ratio (WHR) (one station each), and c) peak expiratory flow (PEF) (L/min)and exhaled carbon monoxide (CO) levels (ppm) (one station each).

For expediency, each respondent sat at a desk and first provided an automated lance finger-prick blood sample onto a test strip read on a Cardiocheck P.A. Analyzer (PTS Diagnostics, Indianapolis) to provide total cholesterol, LDL and glucose levels. Diastolic and systolic blood pressure was next measured with the seated patient’s arm supported on a desk at mid-chest height using an HEM7200 automated blood pressure monitor (Omron Healthcare Ltd., Japan). For anthropometry, workers removed boots, tool-belt and any protective equipment and BMI was automatically calculated using a TPRO5400 Professional BMI machine (Terraillon, Croissy sur Seine) integrated weight/height machine. The weight status of Chinese participants and participants of other nationalities (including Nepalese) was defined based on these measurements in accordance to the WHO recommended BMI classifications for Asians [15] and the WHO recommended BMI classifications for Western populations [15], respectively. Please refer to **Box B in** S1 Appendix for more details. WHR was assessed in centimeters using a 1.5m tape measure positioned 1cm above the iliac crest inside against the skin (waist) and at the widest point of the hip and buttock extension after removal of pocket contents (hip). PEF was measured using a Mini-Wright Peak flow meter (Clement Clark Int, Harlow, UK) with the highest flow of three attempts taken as the final reading. We measured exhaled carbon monoxide using the recommended protocol of inhaling, holding the breath for 15 seconds then exhaling continuously into the intake tube on a PiCo+ CO analyzer (Bentford Scientific Ltd, Maidstone).

Respondents also self-completed an individual standardized health assessment questionnaire addressing basic demographic, socioeconomic, lifestyle and behaviour data. Specifically, respondents were asked about their tobacco and alcohol use, level of work-related physical activity (the amount of physical movement perceived to be involved in respondents’ work for the majority of the time) and leisure activity (amount of exercise that respondents’ engaged in during their free time when they are not at work estimated by the weekly frequency of engaging in such exercise that made participants sweat and breathe somewhat harder than normal in the past 30 days. They were also asked about usual dietary pattern of meats (red and processed) fruits, and vegetables (for which portion examples were provided for quantification purposes). Please refer to **Box A in** S1 Appendix for more details. To maximize response accuracy and data completeness, research assistants provided assistance for questionnaire completion when translations (e.g. Nepalese, Mandarin) or clarification was necessary while respondents queued for the health educator.

Each respondent carried a data sheet for recording all blood, pulmonary, anthropometric and lifestyle data. Once all assessments were completed, respondents queued to see a health educator who provided personalized feedback on the results and interpretation of respondents’ data. Interpretation was based on standardized cardiovascular and pulmonary risk assessment protocols (see ‘Methods‘). Participants’ health data were graphically recorded on a pre-printed personalized health summary card depicting the cardiopulmonary risk profile that each worker retained for reference. Participants with high-risk profiles were advised to consult their respective worksite nurse for confirmatory testing of any suspected raised parameter, who may then refer the participant to seek professional help where necessary.

Please refer to **Box A in** S1 Appendix for details of lifestyle (alcohol consumption, smoking, work-related or leisure time physical activity, fruit and vegetable intake, red and processed meat consumption) and cardiopulmonary (blood pressure, total and HDL blood cholesterol levels, blood glucose, weight status, waist-to-hip ratio, exhaled carbon monoxide level and peak expiratory flow values) risk classifications.

### Statistical analyses

Cardiopulmonary risk and lifestyle profiles of construction workers and office clerks/professionals were differentiated using descriptive analyses with χ^2^-test for categorical and Student’s t-test for continuous variables. The response options for various questionnaire items were collapsed due to small percentages recorded. Multinomial logistic regression was conducted to assess the cross-sectional association between occupational subgroup and number of metabolic risk factors. Simple models adjusted for age and sex. Educational attainment and marital status were additionally adjusted for in a more complex model. All analyses were performed using IBM SPSS (version 20).

### Ethical considerations

Participation was voluntary. All participants of the WHM were employees of, and released by site contractors before each session. All provided written fully-informed consent to participate. Given that the WHM aimed to benefit a maximum number of workers, no exclusion criteria were set. The study protocol was approved by the Institutional Review Board of the University of Hong Kong/ Hospital Authority Hong Kong West Cluster (IRB reference number: UW 14–416).

## Results

Data were collected from 1,903 infrastructure project employees. The work role of 138 participants was not recorded, leaving data on 1,443 frontline construction workers and 322 office clerical/professionals for analysis. The lifestyle characteristics of the clerical and professional office workers were comparable and were thus grouped for analysis.

Most participants were male (81.0%) and Chinese (80.9%) (Table 1). Compared with clerks/professionals, a higher percentage of construction workers were male (83.2% vs. 71.2%), Nepalese (17.8% vs. 1.6%) and were either married or cohabited (81.3% vs. 43.1%) (all *p*<0.001). Construction workers were on average older (mean age 44.9 vs. 35.0 years, *p*<0.001) than clerks/professionals, with up to 39.0% of construction workers aged 50 years or above (data not shown). Construction workers were also less likely to have received tertiary level education (6.1% vs. 72.6%, *p*<0.001) than office clerks/professionals. Most construction workers resided in public rental housing (57.1%) and the monthly domestic household income of most (65.6%) workers did not exceed HKD$25,000.

**Table 1:** Basic socio-demographic characteristics of participants, by occupation subgroup (n = 1765).

Socio-demographic characteristic	Total	Construction workers	Office clerks/ professionals	p for difference between occupation groups^a^
	(n = 1765)	(n = 1443)	(n = 322)	
	n(%)	n(%)	n(%)	
**Gender**				<0.001
Male	1418 (81.0)	1190 (83.2)	228 (71.2)	
Female	332 (19.0)	240 (16.8)	92 (28.8)	
**Age (years)**				<0.001
Mean, SD	43.1 (11.9)	44.9 (11.5)	35.0 (10.7)	
**Ethnicity**				<0.001
Chinese	1424 (80.9)	1134 (78.7)	290 (90.9)	
Nepalese	262 (14.9)	257 (17.8)	5 (1.6)	
Others	74 (3.4)	50 (3.5)	24 (7.5)	
**Educational attainment**				<0.001
Primary or below	443 (25.2)	437 (30.5)	6 (1.8)	
Secondary	991 (56.5)	910 (63.4)	81 (25.3)	
Tertiary or above	321 (18.3)	88 (6.1)	233 (72.6)	
**Monthly domestic household income (HKD$)**				<0.001
<4,000	27 (1.6)	21 (1.5)	6 (1.9)	
4,000- <8,000	33 (1.9)	32 (2.3)	1 (0.3)	
8, 000- <15,000	329 (19.2)	291 (20.8)	38 (12.0)	
15,000- <25,000	634 (36.9)	575 (41.0)	59 (18.6)	
25,000- <40,000	466 (27.1)	373 (26.6)	93 (29.3)	
40,000- <60,000	156 (9.1)	83 (5.9)	73 (23.0)	
≥60, 000	73 (4.2)	26 (1.9)	47 (14.8)	
**Housing condition**				<0.001
Public rental housing	900 (51.6)	815 (57.1)	85 (26.8)	
Subsidized sale flats	180 (10.3)	148 (10.4)	32 (10.1)	
Private permanent housing	462 (26.5)	283 (19.8)	179 (56.5)	
Others	202 (11.6)	181 (12.7)	21 (6.6)	
**Marital status**				<0.001
Never married	377 (21.4)	201 (14.0)	176 (55.0)	
Cohabited/ Married	1307 (74.3)	1169 (81.3)	138 (43.1)	
Widowed	12 (0.7)	11 (0.8)	1 (0.3)	
Divorced/ Separated	60 (3.4)	55 (3.8)	5 (1.6)	
Others	2 (0.1)	2 (0.1)	0 (0.0)	

^a)^ Chi-square test for categorical variables; students t-test used for continuous variables to test for statistical significance of differences observed between occupation groups (construction workers vs. office clerks/professionals) accounting for linear-by-linear association for ordinal variables

Construction workers were also more likely than were clerks/professionals to be daily smokers (39.1% vs. 18.2%) (*p*<0.001), engage in moderate, high or very high levels of work-related physical activity (92.2% vs. 49.0%)(*p*<0.001) and consume fruit (40.1% vs. 28.5%) and vegetables (63.7% vs. 82.7%) (both *p*<0.01) at least 6 days per week (Table 2). Similar proportions of construction workers and clerks/professionals consumed red (34.5% and 35.7%) and processed meat (6.2% and 6.6%) nearly every day. No significant difference was observed for reported alcohol consumption between the two occupational groups.

**Table 2:** Lifestyle characteristics of participants, by occupation subgroup (n = 1765).

Lifestyle behaviour^a^	Total	Construction workers	Office clerks/ professionals
	(n = 1765)	(n = 1443)	(n = 322)
	n(%)	n(%)	n(%)
Smoking pattern***			
*Daily Smoker*	617 (35.3)	559 (39.1)	58 (18.2)
*Occasional Smoker*	41 (2.3)	34 (2.4)	7 (2.2)
*Ex-Smoker*	131 (7.5)	103 (7.2)	28 (8.8)
*Never smoker*	957 (54.8)	732 (51.3)	225 (70.8)
**Alcohol consumption behaviour**			
*High risk drinking (AUDIT-C* ***US*** *positive)*	517 (30.0)	422 (30.0)	95 (30.2)
*Low risk drinking (AUDIT-C* ***US*** *negative)*	1204 (70.0)	984 (70.0)	220 (69.8)
Work-related physical activity level***			
*Very low/ low*	271 (15.6)	111 (7.8)	160 (51.0)
*Moderate*	1019 (58.5)	893 (62.6)	126 (40.1)
*High/very high*	451 (25.9)	423 (29.6)	28 (8.9)
Frequency of leisure time physical activity (per week)***			
*<Once*	955 (55.3)	819 (58.1)	136 (42.6)
*Once*	304 (17.6)	229 (16.3)	75 (23.5)
*2 to 3 times*	249 (14.4)	183 (13.0)	66 (20.7)
*4 to 6 times or ≥once per day*	220 (12.7)	178 (12.6)	42 (13.2)
Days of fruit consumption (per week)**			
*≤1 day*	271 (15.4)	209 (14.6)	62 (19.5)
*2–3 days*	511 (29.2)	412 (28.8)	99 (31.0)
*4–5 days*	304 (17.4)	237 (16.6)	67 (21.0)
*6–7 days*	665 (38.0)	574 (40.1)	91 (28.5)
Days of vegetable consumption (per week)*			
*≤1 day*	84 (4.8)	69 (4.9)	15 (4.7)
*2–3 days*	253 (14.5)	201 (14.0)	52 (16.3)
*4–5 days*	333 (19.0)	249 (17.4)	84 (26.3)
*6–7 days*	1080 (61.7)	912 (63.7)	168 (52.7)
Number of fruits eaten on a day when fruit is consumed**			
*<1 fruit*	344 (19.7)	270 (18.9)	74 (23.3)
*1–2 fruits*	1134 (64.9)	924 (64.7)	210 (66.0)
*3–4 fruits*	184 (10.5)	161 (11.3)	23 (7.2)
*≥5 fruits*	84 (4.8)	73 (5.1)	11 (3.5)
Amount of vegetable eaten on day when vegetable is consumed (bowls)*			
*<1*	332 (19.0)	289 (20.3)	43 (13.5)
*1–2*	1079 (61.9)	875 (61.4)	204 (63.9)
*3–4*	232 (13.3)	176 (12.4)	56 (17.6)
*≥5*	101 (5.8)	85 (6.0)	16 (5.0)
Frequency of red meat consumption (per week)*			
*≤1 day*	201 (11.5)	173 (12.1)	28 (8.8)
*2–3 days*	547 (31.3)	463 (32.4)	84 (26.3)
*4–5 days*	393 (22.5)	300 (21.0)	93 (29.2)
*6–7 days*	608 (34.8)	494 (34.5)	114 (35.7)
Frequency of processed meat consumption (per week)***			
*≤1 day*	1046 (60.5)	911 (64.6)	135 (42.3)
*2–3 days*	431 (24.9)	310 (22.0)	121 (37.9)
*4–5 days*	144 (8.3)	102 (7.2)	42 (13.2)
*6–7 days*	108 (6.2)	87 (6.2)	21 (6.6)

^a)^ Chi-square test to test for statistical significance of differences observed between occupation groups (construction workers vs. office clerks/professionals) accounting for linear-by-linear association for ordinal variables

*p<0.05;

** *p*<0.01;

*** *p*<0.001

More construction workers than clerks/professionals were at higher risk from smoking (48.7% vs. 29.2%) (*p*<0.001) but at low risk in terms of work-related physical activity (92.2%, *p*<0.001), sufficiency of fruit consumption (38.0%, *p*<0.001), red meat (63.3%, *p*<0.001) and processed meat (86.6%, *p*<0.01) consumption patterns (Table 3). There was no significant difference in risk between the two occupation groups in terms of reported alcohol consumption patterns, frequency of leisure time physical activity or sufficiency of vegetable consumption.

**Table 3:** Lifestyle risk groups, by occupation subgroup (n = 1765).

	Total	Construction workers	Office clerks/ professionals
	(n = 1765)	(n = 1443)	(n = 322)
	n(%)	n(%)	n(%)
Smoking pattern***			
***High risk*** *(Current*, *occasional*, *ex-smokers)*	789 (45.2)	696 (48.7)	93 (29.2)
***Low risk*** *(Never smokers)*	957 (54.8)	732 (51.3)	225 (70.8)
**Alcohol consumption behaviour**			
***High risk drinking*** *(AUDIT-C* ***US*** *positive)*	517 (30.0)	422 (30.0)	95 (30.2)
***Low risk drinking*** *(AUDIT-C* ***US*** *negative)*	1204 (70.0)	984 (70.0)	220 (69.8)
Work-related physical activity level***			
***High risk*** *(Very low/ low)*	271 (15.6)	111 (7.8)	160 (51.0)
***Low risk*** *(High/very high/moderate)*	1470 (84.4)	1316 (92.2)	154 (49.0)
**Weekly frequency of leisure time physical activity**			
***High risk*** *(≤Once*, *2 to 3 times)*	1508 (87.3)	1231 (87.4)	277 (86.8)
***Low risk*** *(4 to 6 times*, *>Once per day)*	220 (12.7)	178 (12.6)	42 (13.2)
Weekly fruit consumption***			
***High risk***	1120 (64.0)	887 (62.0)	233 (73.0)
***Low risk*** *(6–7 days/week and 2 fruits/day)*	629 (36.0)	543 (38.0)	86 (27.0)
**Weekly vegetable consumption**			
***High risk***	1577 (90.4)	1296 (90.9)	281 (88.1)
***Low risk*** *(6–7 days/week and 3 servings/day)*	168 (9.6)	130 (9.1)	38 (11.9)
Weekly red meat consumption***			
***High risk***	703 (40.6)	519 (36.7)	184 (58.2)
***Low risk***	1027 (59.4)	895 (63.3)	132 (41.8)
Weekly processed meat consumption**			
***High risk***	252 (14.6)	189 (13.4)	63 (19.7)
***Low risk***	1477 (85.4)	1221 (86.6)	256 (80.3)

*p<0.05;

** *p*<0.01;

*** *p*<0.001

Construction workers were more likely than were clerks/professionals to be hypertensive (22.6% vs. 15.4%, *p*<0.001), have undesirable levels of HDL cholesterol (41.6% vs. 35.8%, *p*<0.05), a diabetic level of non-fasting blood glucose (4.3% vs. 1.6%, *p*<0.05), be overweight or obese (71.7% vs. 56.6%, *p*<0.001) and be centrally obese (53.1% vs. 35.5%, *p*<0.001) (Table 4). Construction workers also had poorer lung function indicated by more having a high risk level of exhaled CO (58.8% vs. 51.3%, *p*<0.001) and a below average peak expiratory flow level (28.2% vs. 22.5%, *p*<0.05).

**Table 4:** Health risk factors, by occupation subgroup (n = 1765).

	Total	Construction workers	Office clerks/professionals
	(n = 1765)	(n = 1443)	(n = 322)
	n(%)	n(%)	n(%)
Blood pressure***			
***Normal*** *(SBP<120 and DBP<80 mmHg)*	256 (14.7)	189 (13.3)	67 (21.0)
***Pre-hypertension*** *(SBP120-139 or DBP 80–89 mmHg)*	1117 (64.0)	914 (64.1)	203 (63.6)
***Hypertension*** *(SBP 140–159 or DBP 90–99 mmHg)*	371 (21.3)	322 (22.6)	49 (15.4)
**Total cholesterol**			
***Desirable*** *(<5*.*2mmol/L)*	1381 (80.5)	1132 (80.6)	249 (80.3)
***Borderline high*** *(≥5*.*2 and <6*.*2mmol/L)*	261 (15.2)	217 (15.4)	44 (14.2)
***High*** *(≥6*.*2mmol/L)*	73 (4.3)	56 (4.0)	17 (5.5)
HDL cholesterol*			
***Undesirable*** *(<1*.*03mmol/L for men*, *<1*.*29mmol/L for women)*	694 (40.5)	583 (41.6)	111 (35.8)
***Acceptable***	641 (37.4)	528 (37.6)	113 (36.5)
***Desirable*** *(≥1*.*55mmol/L; protective against heart disease)*	378 (22.1)	292 (20.8)	86 (27.7)
Blood glucose*			
***Normal*** *(<7*.*8mmol/L)*	1617 (92.3)	1310 (92.4)	307 (97.2)
***Impaired*** *(≥7*.*8 to <9*.*0mmol/L)*	50 (2.9)	46 (3.2)	4 (1.3)
***Diabetic*** *(≥9*.*0mmol/L)*	66 (3.8)	61 (4.3)	5 (1.6)
Weight status***			
***Underweight***	31 (1.8)	22 (1.5)	9 (2.8)
***Normal weight***	505 (28.6)	378 (26.2)	127 (39.4)
***Overweight***	401 (22.7)	334 (23.1)	67 (20.8)
***Obese***	807 (46.3)	694 (48.6)	113 (35.8)
Waist to hip ratio (WHR)***			
***At risk*** *(≥0*.*9 for men; ≥0*.*8 for women)*	867 (49.9)	754 (53.1)	113 (35.5)
***Normal*** *(<0*.*9 for men; <0*.*8 for women)*	872 (50.1)	667 (46.9)	205 (64.5)
Exhaled CO***			
***Non-smoker/normal*** *(0 to 6ppm)*	742 (42.6)	588 (41.2)	154 (48.7)
***Smoker/at risk*** *(>6ppm)*	1000 (57.4)	838 (58.8)	162 (51.3)
Peak expiratory flow (PEF) value*			
***Low PEF (high risk)***	468 (27.2)	397 (28.2)	71 (22.5)
***Normal PEF***	1255 (72.8)	1011 (71.8)	244 (77.5)

*p<0.05;

** *p*<0.01;

*** *p*<0.001

A total of 35% and 18% of clerks/professionals and construction workers, respectively, had no metabolic syndrome risk factors. Concurrently, fewer (9.7%) clerks/professionals than construction workers (12.6%) had 3 or more risk factors for metabolic syndrome. Regression results show that construction workers were more likely than were Clerks/professionals to have 1 (1.87; 95%CI: 1.33 to 2.63), 2 (1.79; 1.25 to 2.55) or 3 (1.77; 1.12 to 2.80) metabolic risk factors than none (Model 1a, Table 5). Observed effects were attenuated once educational attainment and marital status were accounted for in model 1b such that construction workers were still more likely to have 1 metabolic risk factor than office clerks/professionals (1.62; 95%CI: 1.01 to 2.38) but effects for 2 or above risk factors were statistically non-significant.

**Table 5:** Cross-sectional association between occupation subgroup and number of metabolic risk factors (n = 1765).

	Number of metabolic risk factors					
	*Model 1a*^a^			*Model 1b*^b^		
Occupation subgroup	1 (vs. 0) risk factor	2 (vs. 0) risk factors	3 (vs. 0) risk factor	1 (vs. 0) risk factor	2 (vs. 0) risk factors	3 (vs. 0) risk factor
*Office clerks/ professionals*	1	1	1	1	1	1
*Construction workers*	1.87***	1.79**	1.77*	1.62*	1.43	1.63
	(1.33 to 2.63)	(1.25 to 2.55	(1.12 to 2.80)	(1.03 to 2.56)	(0.89 to 2.30)	(0.89 to 2.99)

^a)^ Model 1a adjusted for age and sex

^b)^ Model 1b adjusted for variables in model 1a and additionally for educational attainment and marital status

*p<0.05;

** *p*<0.01;

*** *p*<0.001

A follow-up telephone interview was performed one month later on a subsample of 212/277 (follow-up rate 76.5%) participants. Of the respondents contacted, 48.2% reported having subsequently discussed their health status with family members and 52.8% reported changed health habits due to participation in the WHM. Overall, respondents expressed satisfaction towards the WHM (average programme satisfaction score: 8.60 (SD: 1.06) out of 10, higher scores represented higher levels of satisfaction). The total cost was approximately US$12 per worker screened.

## Discussion

### Cardiopulmonary risk profiling

Poor cardiovascular risk profiles have been reported among construction workers in Ireland, India and Germany [5, 6, 16]. These studies reiterated the importance of identifying populations at high risk for future CVD and of implementing onsite CVD prevention programmes [5, 6, 16]. Similar findings are seen among these mostly Chinese local construction industry workers, who differ from office employees. According to the American Heart Association [17, 18], individuals with 3 or more of 5 metabolic risk factors (raised blood triglycerides, HDL cholesterol or fasting glucose, central obesity, hypertension) meet the diagnostic criteria for metabolic syndrome, placing them at higher cardiovascular risk. Though we only measured 4 of these 5 risk factors, after adjustment for age and gender differences, 9.7% office workers and 12.6% construction workers potentially had at least 3 risk factors for metabolic syndrome (MS). This compares to estimates of general population MS prevalence of 15.7% and 14.2% in European men and women respectively [19] and 9.8% (95% CI 9.0–10.6) in Mainland Chinese men and 17.8% (16.6–19.0) in women [20], though more recent studies report sharply higher rates in Mainland Chinese men (35.1%) and women (32.5%) [21]. Thus the construction workers seem to have lower than average levels of metabolic risk relative to the Mainland Chinese population, but comparable to those of Europeans. Construction workers are more likely than office clerks/professionals to have at least 1 metabolic risk factor even after adjusting for educational attainment and marital status and therefore are a priority. Moreover, significant numbers of these construction workers have below population-average PEF values, particularly those older and more frequently engaged in underground work, possibly reflecting some occupational exposure.

### Lifestyle profile

Lifestyle risk factors, particularly those related to smoking, diet and lack of exercise are closely related to CVD [22]. Although our study is cross-sectional and unable to ascertain causality understanding any lifestyle differences between participating construction workers and office clerks/professionals could potentially have useful implications on future CVD prevention strategies implemented in the construction industry [4, 23].

#### Smoking

Smoking among construction workers is nearly 2 times the rate seen among office clerks/professionals and 4 times the Hong Kong population average (10.7%) [24]. This high smoking prevalence in construction workers coincidental with exhaled CO detected in more than half of the workers, and below population average PEF values in one third of the workers, indicates compromised lung function. Smoking is a key CVD risk factor [22], compromising physical health and causing disability-related premature retirement [25] with significant social and economic consequences. Although intervention is necessary, circumstances make smoking a difficult behaviour to tackle among construction workers. For example, the existing smoking areas at construction worksites do not promote an anti-smoking culture. With this, perhaps the local construction industry needs to make reference to comprehensive plans focused on tobacco control at construction worksites that are implemented elsewhere in the world, such as the BUILT project in California [26]. Possible strategies include on-worksite smoking bans and employer-provided smoking cessation services. Given many non-smokers also had high exhaled CO levels (37.4%) and subpar PEF values (24.5%), worksite tobacco control strategies would also benefit the non-smoking population by lowering levels of second-hand smoking exposure.

#### Alcohol consumption

Although high risk alcohol consumption did not differ between construction workers and office clerks/professionals, about one-in-three from each occupation subgroup met criteria for high risk drinking. This is potentially dangerous given the hazardous nature of construction worksites. If drinking occurs before or during working hours, the risks of accidents are high. Though we did not have information about time of alcohol consumption, among construction workers alcohol is commonly consumed during work breaks [27]. Furthermore, there is evidence that construction workers have low levels of awareness regarding the effects of alcohol on job performance [28]. Hence, apart from softer interventions such as education about the harms of worksites drinking, tougher interventions such as the introduction of mandatory onsite alcohol testing and monitoring should be considered.

#### Dietary habits

A higher percentage of construction workers report having healthier diets featuring higher fruit intake but lower red and processed meat consumption. Concurring with evidence showing that marital status affects health and quality of life [29, 30], married/cohabited participants were more likely to report healthier fruit and, red and processed meat intakes than their never married counterparts (data not shown). Still, most screened respondents consumed insufficient fruits and vegetables while over a third consume more red meat than recommended, increasing their CVD risk. Other than nutrition education and counseling to encourage healthier diets among construction industry employees [23], providing nutritional food in worksite canteens could be beneficial. However, few of these Hong Kong worksites have canteens, and most workers buy oily, salty “meat’n’carb” lunchboxes from nearby retailers.

#### Activity

Construction worker generally involves high levels of physical activity compared with office-work. Current physical activity recommendations seldom differentiate work- from leisure-time physical activity [31], suggesting comparability. However, high physical workload may make fail to improve aerobic capacity or physical fitness volume among employees for whom intensity or duration of physical work is usually insufficient to induce beneficial musculoskeletal or cardiorespiratory effects [32]. Furthermore, higher work-related physical activity may increase risk of CVD-related events [33, 34]. Hence, physical work demands and physical exercise may not be comparable for cardiovascular health [35]. Different definitions exist for physical activity and exercise [36]. Therefore, clarification is needed for the potentially different contributions of occupational physical activity and leisure time exercise towards cardiovascular health. This is particularly important given that many construction workers potentially meet the criteria for MS despite working in a physically demanding industry. Meanwhile, given that close to 90% of both construction and office workers seldom engage in leisure-time exercise, interventions to increase exercise though desirable will be challenging, particularly among construction workers who already consider themselves as active during work.

### Implications

Workplace health risk assessments are important for healthy workplaces [1] particularly for construction workforce where health literacy is likely low and health-related misconceptions are prevalent [2]. This study demonstrated the feasibility of an inexpensive workplace cardiopulmonary risk-screening programme for underserved Hong Kong construction workers. Construction workers frequently involved in physically demanding work and exposed to poor work conditions may not be well-educated, be poorly informed about health risks and would probably not have sought healthcare elsewhere are likely to have benefited the most. Similar objective health risk assessments carried out at worksites minimizing participants’ work schedule disruption conducted on a regular basis would be beneficial in raising health awareness. Identifying employees at high cardiopulmonary risk is an important aim of worksite lifestyle-based CVD prevention interventions targeting improved worker health and performance [4, 23]. Importantly, this study identified different risk and lifestyle profiles for frontline construction workers and office clerks/professionals, implying the need to develop separate health promotion initiatives targeted at employees in different segments of the same work industry.

### Limitations

The study has several limitations. First, though participation was limited to railway infrastructure-contract employees, the wide scope of their work means they are very representative of construction workers, so the results are likely applicable to construction workers in, for example the housing industry.

Second, all sessions were carried out in real-world construction environments, often during inclement weather and high temperature and humidity with very limited resources and support. Thus, we were unable to introduce a prior resting period for blood pressure measurement probably overestimating hypertension prevalence. Still, some workers had clearly abnormal pressure. The appropriateness of BMI as a body weight indicator for muscular construction workers is moot. Since BMI does not differentiate between muscle and fat mass, the prevalence of overweight/obese participants could have been overestimated. However, as a complementary measurement, WHR that indicates central adiposity unaffected by lean mass was also taken. PEF assessment involved at least two ‘blows” for each participant, but ensuring adequate technique was difficult possibly misclassifying some below-average PEF values. Blood glucose and cholesterol levels were assumed to be non-fasting post-prandial levels although we were unable to ascertain when each participant ate their last meal. Nevertheless, this was a screening, not a diagnostic programme where individuals identified as being potentially at high risk were referred on to site nurses for follow-up.

Third, lifestyle self-report-based questionnaire data are prone to reporting bias. However, any biased reporting would likely have underestimated lifestyle risk prevalence among participants. Content was extracted from widely used, well-established and previously validated questionnaires, including the WHO AUDIT and the Hong Kong Behavioural Risk Factor Surveillance System.

Finally, some questionnaire lifestyle and behaviour items differed from existing literature recommendations complicating risk categorization. However, as categorization aim to produce conservative risk estimates, prevalence data likely under- rather than over-estimation actual prevalence. No local recommendations for hazardous drinking using the AUDIT-C classification are unavailable. Hong Kong has a relatively low prevalence of per capita alcohol consumption among adults (≥15 years of age) compared with other regions/countries with available AUDIT-C cut-off recommendations. Nevertheless, we used lower cut-off values and classified men and women with a total score of ≥4 and ≥3 as hazardous drinkers, respectively, adhering to the US and Australian recommendations.

### Conclusions

Inexpensive workplace health risk assessments can identify employees at potentially high cardiopulmonary risk for onward screening referral. In the local construction workforce multiple cardiopulmonary risk factors were observed in large numbers of respondents. Relevant health promotion interventions that target different employee subgroups in the construction industry may be beneficial.

## Supporting Information

S1 Appendix Classification of lifestyle and cardiopulmonary risk levels. (DOCX)
