# Supplementary material for: Workplace Health Promotion: Assessing the Cardiopulmonary Risks of the Construction Workforce in Hong Kong
Source: PLoS One. 2016 Jan 22;11(1):e0146286. doi: 10.1371/journal.pone.0146286 (PMC4723250; doi:10.1371/journal.pone.0146286)
Supplement: S1 Appendix — (DOCX) [file pone.0146286.s001.docx]

| ***Box A. Classification of lifestyle risk*** | |
| --- | --- |
| Alcohol consumption | Using the AUDIT-C 3-item screening protocol, participants were classified into hazardous drinkers (total score ≥4 and ≥3 for men and women, respectively) or low risk drinkers^1, 2^. |
| Smoking | Current and occasional smokers were considered high risk for CVD while never smokers were at low risk. Although former smokers who have quit for >12 months are generally regarded to be at an ‘ideal’ (low) risk for CVD by the American Heart Association^3^, we did not have information on time since quitting for ex-smokers. Taking a conservative stance, ex-smokers were considered at high risk for CVD. |
| Physical activity | Participants reporting moderate, high or very high levels of work-related physical activity *or* leisure time exercise that made them breathe somewhat harder than normal and sweat on at least 4 times/week were classed as sufficiently active (low CVD risk). The American Heart Association^4^ recommends “at least 30 minutes of moderate-intensity aerobic activity for at least 5 days per week for a total of 150 minutes” for overall cardiovascular health. We lacked information on duration of exercise so based our classification on frequency of leisure time exercise: participants exercising ‘4 to 6 times/week’ or ‘≥1 time/day’ were considered sufficiently active. Because current recommendations do not differentiate between activity *type* (i.e. leisure-time or work-related), participants were considered to have met the physical activity recommendations for ideal cardiovascular health if sufficiently active during work *or* leisure-time. |
| Fruit intake | Participants had low CVD risk if they consumed fruit on 6 or 7 days per week *and* had at least 2 servings of fruit each day^5^, else high risk. |
| Vegetable intake | Participants had low CVD risk if they consumed vegetables on 6 or 7 days per week *and* had at least 3 servings of vegetables each day^5^, else high risk. |
| Red meat consumption | World Cancer Research Fund/American Institute for Cancer Research recommendations^6^: limit red meat consumption (daily average x frequency of days) to no more than 500g. We had consumption frequency data available (average days/week) and assumed each day’s red meat intake at the average Hong Kong daily intake among community dwelling people aged 18 to 64^7^. We multiplied the prior by the latter to calculate total weekly amount. |
| Processed meat consumption | We only had information on days/week of processed meat consumption. We were unable to find information on the average daily processed meat consumption of the Hong Kong population. Hence, we assumed 1 serving/day. American Heart Association Guidelines recommend “a modest intake” of processed meat^8^ e.g. <4 servings per week. Hence, we classed workers reporting ≤3 servings/week at low CVD risk |

**S1 APPENDIX: Classification of lifestyle and cardiopulmonary risk levels**

The definition used for the classification of lifestyle and cardiopulmonary risk levels are shown in Box 1 and Box 2, respectively.

| ***Box B. Classification of cardiopulmonary risk levels*** | |
| --- | --- |
| Blood pressure | Following American Heart Association recommendations^9^, BP was categorized as normal (*SBP<120 and DBP<80 mmHg*), pre-hypertension (*SBP120-139 or DBP 80-89 mmHg*) and hypertension *(SBP 140-159 or DBP 90-99 mmHg or higher*). |
| Total blood cholesterol | Total blood cholesterol level was categorized as desirable (*<5.2mmol/L*), borderline high (*≥5.2 and <6.2mmol/L*) or high (*≥6.2mmol/L*) levels^10^. |
| HDL cholesterol level | HDL cholesterol level was categorized as undesirable (*major heart disease risk factor: <1.03mmol/L for men, <1.29mmol/L for women),* acceptable, or desirable *(≥1.55mmol/L; protective against heart disease)* levels^10^*.* |
| Blood glucose | Adapting American Diabetes Association guidelines for blood glucose levels 2 to 3 hours post-prandially^11^, *<7.8mmol/L* was normal, *≥7.8 to <9.0mmol/L* impaired and *≥9.0mmol/L* was considered diabetic *.* |
| Body weight status | We followed WHO recommended BMI classifications for Asians using objectively measured height and weight measurements. (underweight: *<18.5kg/m^2^*; normal weight: *18.5 to <23kg/m^2^*; overweight: *23 to <25kg/*m^2^; obese: *≥25kg/*m^2^) and Western (underweight: *<18.5kg/m^2^*; normal weight: *18.5 to <25kg/m^2^*; overweight: *25 to <30kg/*m^2^; obese: *≥30kg/*m^2^) populations^12^. |
| Waist-to-hip ratio (WHR) | Waist-to-hip ratio was considered normal if <0.9 and <0.8 for men and women, respectively^13^. Individuals with WHR above those values were considered to be centrally obese. |
| Exhaled Carbon Monoxide (CO) level | The level of exhaled carbon monoxide was considered normal if *<6ppm* and high risk for compromised lung function if *≥6ppm* |
| Peak expiratory flow (PEF) values | Values below HK peak flow average for sex and height^14^ indicated high risk of compromised airways |

**REFERENCES**

1. The Royal Australasian College of General Practitioners. Clinical guidelines: AUDIT-C [cited 2014 21st December]. Available from: http://www.racgp.org.au/your-practice/guidelines/redbook/appendices/appendix-3-audit-c/.

2. Bradley KA, DeBenedetti AF, Volk RJ, Williams EC, Frank D, Kivlahan DR. AUDIT‐C as a Brief Screen for Alcohol Misuse in Primary Care. Alcoholism: Clinical and Experimental Research. 2007;31(7):1208-17.

3. Go AS, Mozaffarian D, Roger VL, Benjamin EJ, Berry JD, Borden WB, et al. Heart Disease and Stroke Statistics—2013 Update: A Report From the American Heart Association. Circulation. 2013;127(1):e6-e245.

4. American Heart Association. American Heart Association Recommendations for Physical Activity in Adults 2014 [cited 2014 21st December]. Available from: http://www.heart.org/HEARTORG/GettingHealthy/PhysicalActivity/FitnessBasics/American-Heart-Association-Recommendations-for-Physical-Activity-in-Adults_UCM_307976_Article.jsp.

5. Central Health Education Unit , Department of Health, Government of the Hong Kong SAR. Two Plus Three Every Day - Dietary Recommendations for Fruit and Vegetables Intake 2014 [cited 2014 21st December]. Available from: http://www.cheu.gov.hk/eng/info/2plus3_17.htm.

6. World Cancer Research Fund/American Institute for Cancer Reserach. WCRF/AICR's Second Expert Report: Food, Nutrition, Physical Activity, and the Prevention of Cancer: a Global Perspective. 2007.

7. Centre for Health Protection, Department of Health, Government of the Hong Kong SAR. Red Meat Consumption: the Good and the Bad. Hong Kong: 2012.

8. Mozaffarian D, Appel LJ, Van Horn L. Components of a cardioprotective diet new insights. Circulation. 2011;123(24):2870-91.

9. American Heart Association. Understanding Blood Pressure Readings 2014 [cited 2014 21st December]. Available from: http://www.heart.org/HEARTORG/Conditions/HighBloodPressure/AboutHighBloodPressure/Understanding-Blood-Pressure-Readings_UCM_301764_Article.jsp.

10. American Heart Association. Levels of Cholesterol 2010 [cited 2014 21st December]. Available from: http://www.heart.org/HEARTORG/GettingHealthy/FatsAndOils/Fats101/Levels-of-Cholesterol_UCM_305051_Article.jsp.

11. American Diabetes Association. Standards of medical care in diabetes. Diabetes Care. 2012;35(Supp 1):S12, table 2.

12. World Health Organziation Western Pacific Region. The Asia-Pacific perspective: Redefining obesity and its treatment. 2000.

13. World Health Organization. Waist Circumference and Waist-Hip Ratio: Report of a WHO Expert Consultation (Geneva, 8-11 December 2008). 2008.

14. MSH C, NM W, AYF K, WWS T, YT W, TP L. Nomogram of peak expiratory flow rates (PEFR) for Hong Kong Chinese. HK Pract. 2010;32:120-7.
